# Directional Modulation Technique Using a Polarization Sensitive Array for Physical Layer Security Enhancement

**DOI:** 10.3390/s19245396

**Published:** 2019-12-06

**Authors:** Wei Zhang, Bin Li, Mingnan Le, Jun Wang, Jinye Peng

**Affiliations:** School of Information Science and Technology, Northwest University, Xi’an 710127, China; zhang_wei@nwu.edu.cn (W.Z.); lemingnan@nwu.edu.cn (M.L.); jwang@nwu.edu.cn (J.W.); pjyxida@nwu.edu.cn (J.P.)

**Keywords:** directional modulation, physical layer security, polarization sensitive array, multi-beam

## Abstract

Directional modulation (DM), as an emerging promising physical layer security (PLS) technique at the transmitter side with the help of an antenna array, has developed rapidly over decades. In this study, a DM technique using a polarization sensitive array (PSA) to produce the modulation with different polarization states (PSs) at different directions is investigated. A PSA, as a vector sensor, can be employed for more effective DM for an additional degree of freedom (DOF) provided in the polarization domain. The polarization information can be exploited to transmit different data streams simultaneously at the same directions, same frequency, but with different PSs in the desired directions to increase the channel capacity, and with random PSs off the desired directions to enhance PLS. The proposed method has the capability of concurrently projecting independent signals into different specified spatial directions while simultaneously distorting signal constellation in all other directions. The symbol error rate (SER), secrecy rate, and the robustness of the proposed DM scheme are analyzed. Design examples for single- and multi-beam DM systems are also presented. Simulations corroborate that (1) the proposed method is more effective for PLS; (2) the proposed DM scheme is more power-efficient than the traditional artificial noise aided DM schemes; and (3) the channel capacity is significantly improved compared with conventional scalar antenna arrays.

## 1. Introduction

Wireless communication technologies are increasingly gaining popularity in both military and civil applications due to their inherent flexibility, low installation cost and scalable nature. However, the broadcast nature of the wireless media makes the confidential information transmitted wirelessly vulnerable to interception. Therefore, information security is emerging into a hot research field [1].

Traditionally, the security issues have always been handled by high-layer encryption techniques. However, malicious users can still decrypt the complex cipher text with large amount of computation resources. To this end, researchers have turned their interests towards physical layer security (PLS) as a potential way to complement the traditional cryptographic techniques [2,3]. PLS enables the confidential transmissions by only exploiting the intrinsic properties of a wireless environment, e.g., node spatial distribution [4], aggregate interference [5,6], wireless propagation medium [7,8].

### 1.1. Related Works

Directional modulation (DM), as an emerging PLS technology for wireless communication systems, has attracted lots of attention of researchers over the last decade. The fundamental concept of DM was first introduced in [9], which implies that the modulation happened at the antenna level, instead of at the baseband. DM transmitters using passive parasitic elements [9,10], which rely on the effect of near-field coupling were developed. Due to the complex interactions in the near-field, the DM design process is extremely complicated. Further, the DM techniques using actively driven antenna arrays with reconfigurable phase shifters [11,12,13] or radiators [14] were proposed for simplifying the DM signal synthesis. Based on the same DM structure, Shi H.Z. et al. conducted a detailed study on system parameters, such as element spacing [15], and the quantization of phase shifter [16]. A dual-beam DM technique was introduced in [17], where the I and Q signals are transmitted by two different antennas, such as two four-element arrays or two corner reflector antennas. Antenna subset modulation (ASM) was introduced as an another DM synthesis method in [18], and two extensions of the method based on linear sparse arrays in [19] and the spread-spectrum DM structure with a switched antenna array in [20] were further developed. A DM transmitter using a four-dimensional (4-D) antenna array was proposed by utilizing the time modulation technique in [21]. A DM transmitter using a circular array was proposed in [22] to enhance secrecy performance with the help of the Fourier transform network. In [23], a synthesis-free DM transmitter was realized by using a retro-directive array.

In addition to the above-mentioned single-beam DM approaches with only one pre-specified spatial direction, the multi-beam DM synthesis methods with multiple desired directions have also been extensively investigated in [24,25,26,27,28]. The orthogonal vector approach with the help of artificial noise (AN) for multi-beam DM transmitters synthesis was proposed in [24], which is capable of synchronously projecting multiple independent data streams into different destinations while concurrently scrambling the signals leaking out in other undesired directions. The work in [25] demonstrated a robust DM synthesis method for a multi-beam DM system with imperfect estimations of desired directions. The AN was also applied to achieve multi-beam DM systems by utilizing precoding [26] and iterative convex optimization [27]. Xie T. et al. further proposed the zero-forcing based approach for multi-beam DM synthesis with the help of AN [28]. Hafez M. et al. derived the array weights using the least-norm solution and directionally modulated symbols towards to two different directions in a multi-user multiple-input multiple-output (MU-MIMO) system [29]. Furthermore, Hafez M. et al. utilized the dispersive nature of the channel to provide a location-based secure communication link to highly degrade the wiretap channel [30]. In [31], ASM was used efficiently for multidirectional broadcasting communication and maintaining its inherent security.

However, the polarization information is not taken into account for the works mentioned above. The polarization state (PS), as a source of separation, can be utilized to enhance the data rate to make spectrum efficient. For example, Wei D. et al. used the antennas to produce the PSs, then combined the PSs with amplitude-phase modulation (APM) together with confidential information, which further enhanced the data rate on the basis of the traditional APM [32]. Similarly, a spectrum efficient polarized phase shifting keying/quadrature amplitude modulation (PSK/QAM) scheme in the wireless depolarized channel was proposed in [33]. The authors in [34] proposed the polarization modulation (PM) based on a dual-polarized phased array to enhance PLS in millimeter-wave (mm-wave) communication systems. In order to increase the secure performance of a dual-polarized satellite MIMO system, a directional PM scheme using a four-element array was put forward in [35], which can be viewed as the first amalgamation of DM and PM techniques. Recently, a combination of DM and polarization design using crossed dipole arrays was put forward in [36]. Nevertheless, the polarization information is directionless for all the works mentioned above.

### 1.2. Our Contribution

Inspired by the previous pioneering researches, especially in [30,36], this article proposes a multi-beam DM technique using a polarization sensitive array (PSA) with directional polarization information. The spatial domain and polarization domain are simultaneously introduced into the proposed DM scheme. That can further improve the traditional multi-beam DM systems’ spectrum efficiency. Meanwhile, that also can provide a more secure communication link for transmitted data streams. Overall, the main contributions of the article can be summarized as follows:

(1) Based on a PSA, we realize the multi-beam DM synthesis using a precoding matrix to send independent data streams with different PSs to further increase the channel capacity.

(2) The proposed DM scheme utilizes the directional polarization formation to enhance PLS, which outperforms the conventional multi-beam AN-aided DM schemes in power-efficient.

(3) The symbol error rate (SER), secrecy rate, and robustness of the proposed DM scheme are deduced and corroborated with simulations. Design examples are also provided to verify the effectiveness of the proposed DM method.

The proposed DM technique can be typically applied to the future line-of-sight (LoS) communication networks. The potential application scenarios include future Internet of things (IoT), vehicular communications, smart transportation, military communications, satellite communications, unmanned aerial vehicle (UAV) networks [29], and mm-wave communications [8,37].

The rest of the article is organized as follows: Section 2 presents the system model. Section 3 provides the review of polarized beamforming. Section 4 elaborates the principle of the proposed multi-beam DM technique using a PSA from the perspective of sampling and signal processing, respectively. Section 5 deduces the security performance of the proposed DM scheme, and further compares different multi-beam DM schemes. Section 6 gives simulation results and discussions and Section 7 concludes the article. The notations used in this article is summarized in Table 1.

## 2. System Model

**Figure 1:**
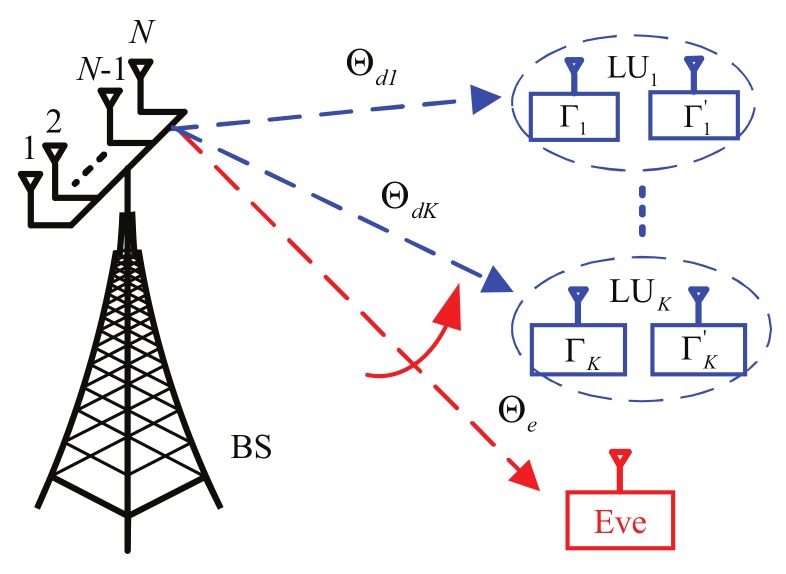
System model.

Consider a multiple-input single-output (MISO) system with *N* transmitting antennas for the base station (BS), (i.e., source transmitter) and one single receiving antenna for a legitimate user (LU) or an eavesdropper (Eve) as depicted in Figure 1. Meanwhile, the BS sends confidential messages to *K* LUs along the LoS paths in the presence of an Eve. Here, the BS has a-priori information about the directions of the *K* LUs, but not of the potential Eve. Specifically, in order to utilize the polarization information, a PSA is adopted for the proposed system. However, the idea proposed in this article can also be extended to a MIMO system for multiple LUs and Eves.

**Figure 2:**
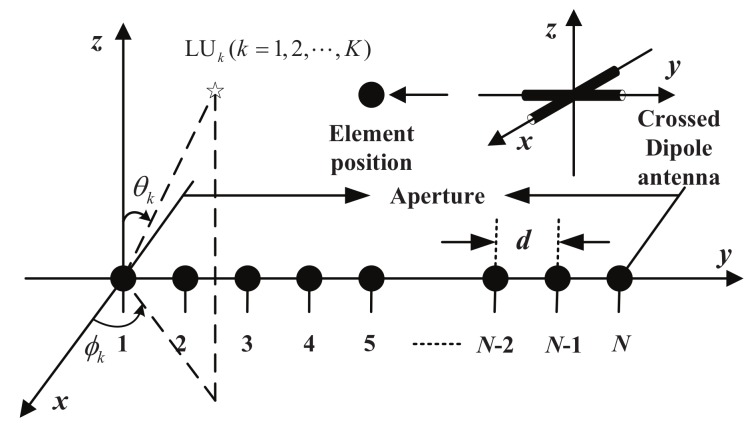
A polarization sensitive array (PSA) structure.

Unless note otherwise, a narrow-band linear PSA is assumed for the BS in this article. A PSA structure with *N* crossed dipole antennas is shown in Figure 2. However, other polarization sensitive antennas also work. The *N* crossed dipole antennas are evenly distributed with an appropriate adjacent spacing distance *d* to avoid spatial aliasing and antenna coupling. For each polarization sensitive antenna, there are two orthogonally oriented dipoles. One is parallel to the *x*-axis and the other is parallel to the *y*-axis. The spacing from the first antenna to itself and its subsequent antennas is represented by $d_{n}$ for *n* = 1, 2, ⋯, *N*. Also shown is a desired LU with its accurate DOA (direction of arrival) defined by the elevation angles θ∈[−ππ22,ππ22] and the azimuth angles ϕ∈[−π,π].

## 3. Review of Polarized Beamforming

In this system, there are *K* different desired directions, and 1≤K<N. A plane-wave model is assumed, i.e., the LUs are located in the far-field region. The spatial steering vector for the given array geometry is expressed as

(1) $$s_{spa}\left(\theta ,\phi \right)=[e^{-j\beta d_{1}\sin\theta \sin\phi },\cdots ,e^{-j\beta d_{n}\sin\theta \sin\phi },\cdots ,e^{-j\beta d_{N}\sin\theta \sin\phi }{]}^{T},$$

where β=2π2πλλ is the intrinsic propagation constant, the parameter λ is the wavelength at the carrier frequency of interest, and $d_{n}$ is given by

(2) $$d_{n}=\left(n-1\right)d,\;\;\;n=1,2,\cdots ,N.$$

Furthermore, for polarization sensitive antennas, the spatial-polarization coherent vector contains spatial information and polarization information of the signal. This vector is given by [38]

(3) $$\begin{array}{cc} s_{pol}\left(\theta ,\phi ,\gamma ,\eta \right) & =\left[\begin{array}{c} -\sin\phi \cos\gamma +\cos\theta \cos\phi \sin\gamma e^{j\eta }\\ \cos\phi \cos\gamma +\cos\theta \sin\phi \sin\gamma e^{j\eta } \end{array}\right]\\ \; & =\left[\begin{array}{cc} -\sin\phi & \cos\theta \cos\phi \\ \cos\phi & \cos\theta \sin\phi \end{array}\right]\left[\begin{array}{c} \cos\gamma \\ \sin\gamma e^{j\eta } \end{array}\right]\\ \; & =\left[\begin{array}{c} s_{pol,H}\left(\theta ,\phi ,\gamma ,\eta \right)\\ s_{pol,V}\left(\theta ,\phi ,\gamma ,\eta \right) \end{array}\right], \end{array}$$

where γ∈[0,ππ22] represents the auxiliary polarization angle and η∈[−π,π) is the polarization phase difference.

The polarization constellation point which denotes a polarization state is given by

(4) $$P_{n}=\left[\begin{array}{c} \cos\gamma_{n}\\ \sin\gamma_{n}e^{j\eta_{n}} \end{array}\right]=\left[\begin{array}{c} w_{Hn}\\ w_{Vn} \end{array}\right].$$

For the sake of description and analysis, we first define

(5) $$(\theta ,\phi ,\gamma ,\eta )\overset{\Delta }{\;=}(\Theta ,\Gamma ),$$

where Θ and Γ denote spatial information and polarization information, respectively. Then, the PSA can be split into two sub-arrays, one parallel to each axis. Thus, the steering vectors of the two sub-arrays are given by

(6) $$\begin{array}{c} s_{H}\left(\Theta ,\Gamma \right)=s_{pol,H}\left(\Theta ,\Gamma \right)s_{spa}\left(\Theta ,\Gamma \right),\\ s_{V}\left(\Theta ,\Gamma \right)=s_{pol,V}\left(\Theta ,\Gamma \right)s_{spa}\left(\Theta ,\Gamma \right). \end{array}$$

Finally, the overall steering vector of the PSA is given by

(7) $$\begin{array}{cc} s(\Theta ,\Gamma ) & =s_{pol}\left(\Theta ,\Gamma \right)⊗s_{spa}\left(\Theta ,\Gamma \right)\\ \; & =\left[\begin{array}{c} s_{H}\left(\Theta ,\Gamma \right)\\ s_{V}\left(\Theta ,\Gamma \right) \end{array}\right]. \end{array}$$

The beam response of the PSA is given by

(8) $$p\left(\Theta ,\Gamma \right)=w^{H}s\left(\Theta ,\Gamma \right),$$

where w, a 2N×1 vector denoting the overall complex valued weight coefficients of the full array, is given by

(9) $$w={\left[w_{1,H},\cdots ,w_{N,H},w_{1,V},\cdots ,w_{N,V}\right]}^{T}.$$

**Figure 3:**
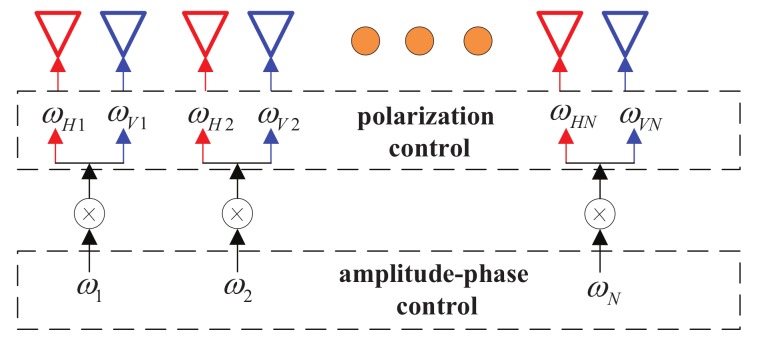
A schematic diagram of the amplitude-phase and polarization independent control.

Figure 3 shows a schematic diagram of the amplitude-phase and polarization independent control algorithm for a PSA. For the polarization control portion, each antenna element is separately weighted firstly. After the element weighting is completed, the array factor is controlled by the classical array factor control method. Then, we have

(10) $$\left\{\begin{array}{c} w_{n,H}=w_{n}\cdot w_{Hn}\\ w_{n,V}=w_{n}\cdot w_{Vn} \end{array}\right.,\;n=1,2,\cdots ,N.$$

## 4. Principle of the Proposed Multi-Beam DM Technique Using a PSA

In this section, we formulate the design to achieve DM for *K* desired directions for the same data stream or independent data streams. Aiming at each desired direction, PM can be adopted to convey independent data streams simultaneously using a PSA. We will propose two methods to achieve multi-beam DM synthesis from the perspective of sampling in the spatial and the polarization domain, and from the perspective of signal processing, respectively.

### 4.1. From a Sampling Perspective

The essence of the DM design for a PSA is to find a set of weight coefficients that give directional responses. In doing so, a certain constellation with low SER can be realized in desired directions, while the constellation will be scrambled in other undesired directions to result in high SER for illegal users.

Take *M*-ary signaling, like multiple phase shift keying (MPSK) for example, there are *M* sets of desired array response across a diversity polarization channel in one anticipant direction. Therefore, for DM across a polarization diversity channel in *K* different desired directions, i.e., there exist *K* beams, the total number of sets of desired response $p_{i}\left(\Theta ,\Gamma \right)$ is given by

(11) $$T=\prod_{j=1}^{K}M_{j},$$

where $M_{j}$ denotes that $M_{j}$-PSK signaling is transmitting in the *j*th direction. Furthermore, each desired response $p_{i}\left(\Theta ,\Gamma \right)$, i=1,2,⋯,T, can be viewed as a function of $\left(\Theta ,\Gamma \right)$.

From the spatial domain prospective, each desired response $p_{i}\left(\Theta ,\Gamma \right)$ can be split into two regions: the spatial mainlobe and the spatial sidelobe according to the azimuthal points of interest or not. Without loss of generality, we assume that *r* (r≥1 ) points are sampled in each desired direction and *R* (R≫1 ) points in the sidelobe region. In order to narrow the information beam width to enhance the security, *r* is always set to be 1, and *R* is usually set to be a large number.

Meanwhile, from the polarization domain prospective, each desired response $p_{i}\left(\Theta ,\Gamma \right)$ can also be split into two regions: the polarization mainlobe and the polarization sidelobe according to the polarization points of interest or not. Like the spatial sampling, we designate fixed PSs for different polarization diversity channels, for all sampled responses in the desired directions. While, the expected responses at different sidelobe directions with different PSs are generated randomly.

Then, the corresponding responses in the mainlobe range and sidelobe region are given by

(12) $$p_{i,ML}=\left[p_{i}\left(\Theta_{1},\Gamma_{1}\right),p_{i}\left(\Theta_{2},\Gamma_{2}\right),\cdots ,p_{i}\left(\Theta_{K},\Gamma_{K}\right)\right],$$

and

(13) $$\begin{array}{c} p_{i,SL}=\left[p_{i}\left(\Theta_{K+1}\Gamma_{K+1}\right),p_{i}\left(\Theta_{K+2}\Gamma_{K+2}\right),\cdots ,p_{i}\left(\Theta_{K+R}\Gamma_{K+R}\right)\right], \end{array}$$

respectively.

Moreover, before proceeding to the next step, we define a 2N×Kr matrix $S_{ML}$ and a 2N×R matrix $S_{SL}$, given by

(14) $$\begin{array}{c} S_{ML}=\left[s\left(\Theta_{1},\Gamma_{1}\right),s\left(\Theta_{2},\Gamma_{2}\right),\cdots ,s\left(\Theta_{K},\Gamma_{K}\right)\right], \end{array}$$

and

(15) $$\begin{array}{c} S_{SL}=\left[s\left(\Theta_{K+1}\Gamma_{K+1}\right),s\left(\Theta_{K+2}\Gamma_{K+2}\right),\cdots ,s\left(\Theta_{K+R}\Gamma_{K+R}\right)\right], \end{array}$$

respectively.

Next, for the *i*th constellation point, its corresponding weight coefficients can be obtained by

(16) $$\begin{array}{c} \min\;\;\;{∥p_{i,SL}-w_{i}^{H}S_{SL}∥}_{2}\\ s.t.\;p_{i,ML}=w_{i}^{H}S_{ML}. \end{array}$$

The objective function enables a minimum difference between the intended and designated responses in the sidelobe for the different polarization diversity channels. And the constraint condition in Equation (16) ensures to maintain desired polarization constellation points in desired directions. In order to enhance the security performance of the system, we have to distort the constellation points in the sidelobe. The amplitude, phase and the polarization information of the desired response at different directions in the sidelobe are all generated at random for every constellation point.

The optimization problem in Equation (16) can be solved by using the method of Lagrange multipliers. The optimal solution for the weight coefficient vector $w_{i}$ can be expressed as

(17) $$\begin{array}{c} w_{i}=R^{-1}\left(S_{SL}p_{{}_{SL,i}}^{H}-S_{ML}{\left(S_{{}_{ML}}^{H}R^{-1}S_{ML}\right)}^{-1}\left(S_{{}_{ML}}^{H}R^{-1}S_{SL}p_{{}_{SL,i}}^{H}-p_{{}_{ML,i}}^{H}\right)\right) \end{array}.$$

The proof is seen in Appendix A.

**Figure 4:**
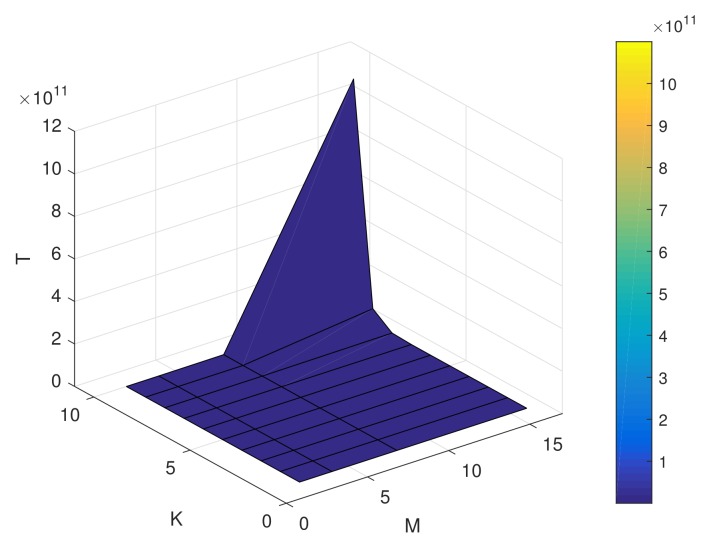
The numbers of the Lagrangian multiplier method computations for the proposed directional modulation (DM) scheme.

In Figure 4, the numbers of the Lagrangian multiplier method computations for the proposed DM scheme is shown. It is easy to observe that the computational complexity is extremely high when the number of the desired directions and the modulation order are large. Therefore, in a practical system, there is a tradeoff between the computational complexity, the number of the desired directions, and the modulation orders for performance gain.

### 4.2. From a Signal Processing Perspective

**Figure 5:**
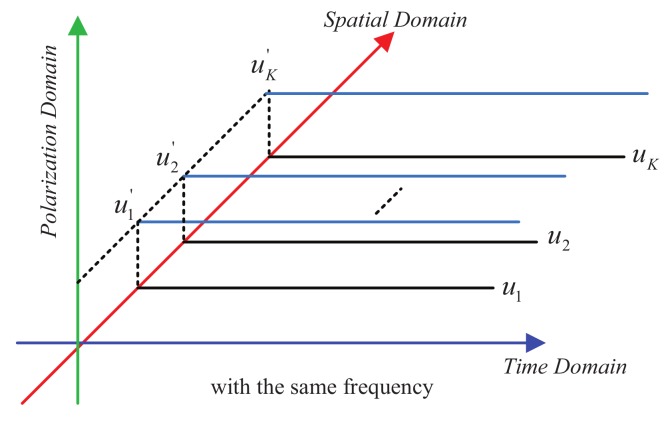
A schematic diagram of joint the time-polarization-spatial domain.

As we all know, besides the carrier’s amplitude and phase, the carrier’s PS can also be used as the information bearing parameter, which can further improve the conventional DM methods’ spectrum efficiency. A schematic diagram of joint the time-polarization-spatial domain is shown in Figure 5. Each direction can have two desired data streams with the same or different modulation modes by using different PSs, and also sharing the same resources of time slots, frequency bands. Therefore, the spectrum efficiency is significantly improved in our methods.

Due to the introduction of polarization information, we can consider a scenario where the BS is trying to transmit confidential messages to *K* different directions, and each direction with two different PSs, as shown in Figure 1. For clarity, only one PS is analyzed in this part. Assuming that the BS has a-priori knowledge of the desired directions and polarization information of all LUs. Before transmitting, the data streams should be processed with a pre-coding matrix P to match the *N* transmit antennas. In order to obtain the precoding matrix P, the spatial positions and polarization information of all LUs are specified, the combined set of which can be expressed as

(18) $$\begin{array}{c} \left(\Theta^{\mathrm{LU}},\Gamma^{\mathrm{LU}}\right)=\left\{\left(\Theta_{1}^{\mathrm{LU}},\Gamma_{1}^{\mathrm{LU}}\right),\cdots ,\left(\Theta_{k}^{\mathrm{LU}},\Gamma_{k}^{\mathrm{LU}}\right),\cdots ,\left(\Theta_{K}^{\mathrm{LU}},\Gamma_{K}^{\mathrm{LU}}\right)\right\}, \end{array}$$

where $\left(\Theta_{k}^{\mathrm{LU}},\Gamma_{k}^{\mathrm{LU}}\right)$, k∈{1,2,⋯,K}, is the specified spatial direction and polarization information of the *k*th LU. Then, the steering vectors of all LUs can compose a steering matrix, i.e.,

(19) $$\begin{array}{c} H\left(\Theta^{\mathrm{LU}},\Gamma^{\mathrm{LU}}\right)=\left\{s\left(\Theta_{1}^{\mathrm{LU}},\Gamma_{1}^{\mathrm{LU}}\right),\cdots ,s\left(\Theta_{k}^{\mathrm{LU}},\Gamma_{k}^{\mathrm{LU}}\right),\cdots ,s\left(\Theta_{K}^{\mathrm{LU}},\Gamma_{K}^{\mathrm{LU}}\right)\right\}. \end{array}$$

Using the steering matrix in Equation (19), the precoding matrix P at the BS can be designed as [39,40]

(20) $$\begin{array}{c} P=[H\left(\Theta^{\mathrm{LU}},\Gamma^{\mathrm{LU}}\right){]}^{+}=H\left(\Theta^{\mathrm{LU}},\Gamma^{\mathrm{LU}}\right){\left[H^{H}\left(\Theta^{\mathrm{LU}},\Gamma^{\mathrm{LU}}\right)H\left(\Theta^{\mathrm{LU}},\Gamma^{\mathrm{LU}}\right)\right]}^{-1}, \end{array}$$

which is normalized to the steering matrix, i.e.,

(21) $$\begin{array}{c} H^{H}\left(\Theta^{\mathrm{LU}},\Gamma^{\mathrm{LU}}\right)P=I_{K}. \end{array}$$

The radiating signal $x={\left[x_{1},\cdots ,x_{N}\right]}^{T}$ after precoding for the *N* dipole antenna elements can be obtained by

(22) $$\begin{array}{c} x=\sqrt{P_{t}}\mathrm{Pu}, \end{array}$$

where $u=[u_{1},u_{2},\cdots ,u_{K}{]}^{T}$ is the transmitting symbol vector by the *N* polarization sensitive antennas, i.e., the different data streams, $P_{t}$ is the total transmitting power.

## 5. Security Performance Analysis for the Proposed DM Scheme

In this section, we will firstly analyze the security performance for the proposed DM scheme when Eves with and without polarization information. Then, we will analyze the security performance from a signal signal processing perspective. Finally, we will analyze three measurements, including the SER, secrecy rate, and robustness of the proposed DM scheme.

### 5.1. Security Performance Analysis When Eves with Polarization Information

In this part, assuming that the LUs and Eves all know the used PSs and the path loss is neglected.

The signal vector transmitted by an *N*-element PSA across one polarization diversity channel at discrete time *i* is given by

(23) $$\begin{array}{c} D\left(i\right)={\left[d_{1}\left(i\right),d_{2}\left(i\right),\cdots ,d_{K}\left(i\right)\right]}^{T} \end{array}.$$

Through the LoS channel, the received signal vector **y** is

(24) $$\begin{array}{c} y(\Theta ,\Gamma ,i)=H(\Theta ,\Gamma )W(i)D(i)+\xi , \end{array}$$

where **y** is a K×1 vector representing the received signals, $H={\left[s_{1},\cdots ,s_{j},\cdots ,s_{K}\right]}^{T}$ is a K×2N matrix denoting the channel matrix from transmitter to receiver, $s_{j}$ is a 2N×1 vector denoting the steering vector. $W=[w_{1},\cdots ,w_{j},\cdots ,w_{K}]$ is a 2N×K matrix denoting the antenna weights. The variable ξ with distribution $CN(0,\sigma^{2}I_{K})$ is the normalized additive white Gauss noise (AWGN), where CN denotes a complex and circularly symmetric random variable.

Meanwhile, in order to guarantee that the LUs receive the exact information, the *j*th user should receive the *j*th symbol. Then, letting $x\left(i\right)={\left[x_{1}\left(i\right),\cdots ,x_{j}\left(i\right),\cdots ,x_{K}\left(i\right)\right]}^{T}$, and neglecting the noise, we have

(25) $$\begin{array}{c} x(i)=H(\Theta ,\Gamma )W(i)D(i) \end{array}.$$

Because K≥N, Equation (25) is an overdetermined equation with no exact solution. Therefore, we only consider the scenario K<N, i.e., the number of LUs is less than that of transmit antennas. If the receivers with the same DOA and polarization parameters want to receive different information, and Equation (25) becomes a nonuniform equation with no solution. Therefore, the different receivers with different spatial and polarization parameters are requested. The minimum norm solution for Equation (25), i.e., the signal transmitted by the antenna array, is given by

(26) $$\begin{array}{c} D\left(i\right)={\left({\left(H(\Theta ,\Gamma )W(i)\right)}^{H}H\left(\Theta ,\Gamma \right)W\left(i\right)\right)}^{-1}{\left(H(\Theta ,\Gamma )W(i)\right)}^{H}x\left(i\right) \end{array}.$$

In an arbitrary desired direction $\Theta_{j}$, (j∈{1,2,⋯,K} ), the steering vector can be expressed as

(27) $$\begin{array}{c} H\left(\Theta_{j},\Gamma \right)=e_{j}H\left(\Theta ,\Gamma \right), \end{array}$$

where $e_{j}\in R^{1\times K}$ denotes a unit vector, the *j*th term of which is one.

Then, the received signal for desired receiver is given by

(28) $$\begin{array}{c} y_{j}\left(i\right)=H\left(\Theta_{j},\Gamma \right)D\left(i\right)=x_{j}\left(i\right). \end{array}$$

Therefore, an arbitrary desired receiver can recover the exact confidential information.

For an Eve in the direction $\Theta_{e}$ with a polarization sensitive antenna, the steering vector can be written as

(29) $$H\left(\Theta_{e},\Gamma \right)=\mathrm{rH}\left(\Theta ,\Gamma \right),$$

where $r\in R^{1\times K}$, which is given by

(30) $$\begin{array}{c} r={\left({\left(H(\Theta ,\Gamma )W(i)\right)}^{H}H\left(\Theta ,\Gamma \right)W\left(i\right)\right)}^{-1}{\left(H(\Theta ,\Gamma )W(i)\right)}^{H}H\left(\Theta_{e},\Gamma \right) \end{array}.$$

Then, the received signal for undesired receiver is given by

(31) $$y_{e}\left(i\right)=H\left(\Theta_{e},\Gamma \right)D\left(i\right)=r\cdot x\left(i\right).$$

Assuming that the Eve expects to intercept the confidential information for the *l*th (l∈{1,2,⋯,K} ) LU. For the Eve, the information of other LUs can be regarded as noise interference or artificial interference. Then, the received signal for the eavesdropper can be rewritten as

(32) $$y_{e}\left(i\right)=r_{l}\cdot x_{l}\left(i\right)+\sum_{j=1,j\neq l}^{K}r_{j}\cdot x_{j}\left(i\right).$$

Obviously, the modulated signal $x_{l}\left(i\right)$, that the eavesdroppers intend to crack, is seriously affected by the variables $r_{l}$ and $\sum_{j=1,j\neq l}^{K}r_{j}\cdot x_{j}\left(i\right)$. This results in that Eves cannot demodulate the exact confidential information. Therefore, PLS is enhanced.

### 5.2. Security Performance Analysis When Eves without Polarization Information

Assuming that PM is adopted for information transmission for the *K* LUs using a PSA. Then, we can decompose the signals transmitted into two parts in the polarization domain: the horizontal component ($x_{H}\left(i\right)$ ) and the vertical component ($x_{V}\left(i\right)$ ). They are given by

(33) $$\begin{array}{c} x_{H}\left(i\right)={\left[\cos\gamma_{1,i},\cos\gamma_{2,i},\cdots ,\cos\gamma_{L,i}\right]}^{T}, \end{array}$$

and

(34) $$\begin{array}{c} x_{V}\left(i\right)={\left[\sin\gamma_{1,i}e^{j\eta_{1,i}},\sin\gamma_{2,i}e^{j\eta_{2,i}},\cdots ,\sin\gamma_{L,i}e^{j\eta_{L,i}}\right]}^{T} \end{array},$$

respectively.

When the receiver in the direction Θ uses a polarization sensitive antenna, the channel matrix is given by

(35) $$h=s\left(\Theta ,\Gamma \right)⊗\left[\begin{array}{cc} 1 & 0\\ 0 & 1 \end{array}\right].$$

When the receiver in the direction Θ uses horizontally polarized antenna, the channel matrix is given by

(36) $$h_{H}=s\left(\Theta ,\Gamma \right)⊗\left[\begin{array}{cc} 1 & 0 \end{array}\right].$$

Similarly, when the receiver in the direction Θ uses vertically polarized antenna, the channel matrix is given by

(37) $$h_{V}=s\left(\Theta ,\Gamma \right)⊗\left[\begin{array}{cc} 0 & 1 \end{array}\right].$$

Therefore, the signal vector transmitted can be rewritten as

(38) $$D\left(i\right)=D_{H}\left(i\right)⊗\left[\begin{array}{c} 1\\ 0 \end{array}\right]+D_{V}\left(i\right)⊗\left[\begin{array}{c} 0\\ 1 \end{array}\right],$$

(39) $$\begin{array}{c} D_{H}\left(i\right)={\left({\left(H_{H}W\left(i\right)\right)}^{H}H_{H}W\left(i\right)\right)}^{-1}{\left(H_{H}W\left(i\right)\right)}^{H}x_{H}\left(i\right), \end{array}$$

(40) $$\begin{array}{c} D_{V}\left(i\right)={\left({\left(H_{V}W\left(i\right)\right)}^{H}H_{V}W\left(i\right)\right)}^{-1}{\left(H_{V}W\left(i\right)\right)}^{H}x_{V}\left(i\right), \end{array}$$

where

(41) $$H_{H}={\left[h_{H}\left(\Theta {}_{1},\Gamma_{1}\right),\cdots ,h_{H}\left(\Theta {}_{K},\Gamma_{K}\right)\right]}^{T},$$

(42) $$H_{V}={\left[h_{V}\left(\Theta {}_{1},\Gamma_{1}\right),\cdots ,h_{V}\left(\Theta {}_{K},\Gamma_{K}\right)\right]}^{T}.$$

If the receiver located in the desired direction $\Theta_{j}$ (j∈{1,2,⋯,K} ) uses a polarization sensitive antenna, the received signal can be expressed as

(43) $$\begin{array}{c} E\left(\Theta_{j},i\right)=h\left(\Theta_{j}\right)\cdot D\left(i\right)=\left[\begin{array}{c} s\left(\Theta_{j},\Gamma \right)\cdot D_{H}\left(i\right)\\ s\left(\Theta_{j},\Gamma \right)\cdot D_{V}\left(i\right) \end{array}\right]=\left[\begin{array}{c} \cos\gamma_{j,i}\\ \sin\gamma_{j,i}e^{j\eta_{j,i}} \end{array}\right]. \end{array}$$

Therefore, the *K* LUs can receive the exact signal.

Assuming that an Eve wants to intercept the confidential information for the *l*th (l∈{1,2,⋯,K}) LU. When the Eve does not know what antenna is used by the *l*th user, the eavesdropper receiver is likely to utilize a polarization sensitive antenna or a single polarized antenna. If the receiving antenna used by the Eve is different from the *l*th user, the information intercepted cannot be accurately demodulated.

When the Eve adopts a polarization sensitive antenna to receive the confidential information and does polarization demodulation, the PS received by the Eve can be given by

(44) $$P_{e}=\left[\begin{array}{c} \cos\gamma_{e}\\ \sin\gamma_{e}e^{j\eta_{e}} \end{array}\right],$$

(45) $$\begin{array}{c} \gamma_{e}=\arctan\left(\frac{r_{V}\cdot x_{V}\left(i\right)}{r_{H}\cdot x_{H}\left(i\right)}\right)=\arctan\left(\frac{r_{lV}\cdot x_{lV}\left(i\right)+\sum_{j=1,j\neq l}^{K}r_{jV}\cdot x_{jV}\left(i\right)}{r_{lH}\cdot x_{lH}\left(i\right)+\sum_{j=1,j\neq l}^{K}r_{jH}\cdot x_{jH}\left(i\right)}\right). \end{array}$$

(46) $$\begin{array}{cc} \eta_{e} & =\Xi \left(r_{V}\cdot x_{V}\left(i\right)\right)-\Xi \left(r_{H}\cdot x_{H}\left(i\right)\right)\\ \; & =\Xi \left(r_{lV}\cdot x_{lV}\left(i\right)+\sum_{j=1,j\neq l}^{K}r_{jV}\cdot x_{jV}\left(i\right)\right)-\Xi \left(r_{lH}\cdot x_{lH}\left(i\right)+\sum_{j=1,j\neq l}^{K}r_{jH}\cdot x_{jH}\left(i\right)\right). \end{array}$$

Thereinto,

(47) $$\begin{array}{c} r_{H}=s\left(\Theta ,\Gamma \right){\left({\left(H_{H}W\left(i\right)\right)}^{H}H_{H}W\left(i\right)\right)}^{-1}{\left(H_{H}W\left(i\right)\right)}^{H}, \end{array}$$

(48) $$\begin{array}{c} r_{V}=s\left(\Theta ,\Gamma \right){\left({\left(H_{V}W\left(i\right)\right)}^{H}H_{V}W\left(i\right)\right)}^{-1}{\left(H_{V}W\left(i\right)\right)}^{H}. \end{array}$$

Therefore, the received signal by the Eve is distorted in the polarization domain by the equivalent artificial interference $\sum_{j=1,j\neq l}^{K}\frac{r_{jV}}{r_{lV}}\cdot x_{jV}\left(i\right)$ and $\sum_{j=1,j\neq l}^{K}\frac{r_{jH}}{r_{lH}}\cdot x_{jH}\left(i\right)$. So, it is a very hard task for an Eve to demodulated the useful information without *a*-*prior* knowledge of polarization parameters, and PLS can also be guaranteed.

### 5.3. Security Performance Analysis from a Signal Processing Perspective

In this part, we will analyze the security performance of the proposed DM scheme from a signal processing perspective.

After passing through the LoS channel, the received signal of the *k*th LU is obtained by

(49) $$\begin{array}{c} y_{{}^{k}}^{\mathrm{LU}}=y\left(\Theta_{k}^{\mathrm{LU}},\Gamma_{k}^{\mathrm{LU}}\right)=s^{H}\left(\Theta_{k}^{\mathrm{LU}},\Gamma_{k}^{\mathrm{LU}}\right)x+\xi_{k}^{\mathrm{LU}}, \end{array}$$

where $\xi_{k}^{\mathrm{LU}}∼CN(0,\sigma_{\xi^{\mathrm{LU}}}^{2})$ is the AWGN with zero mean and variance $\sigma_{\xi^{\mathrm{LU}}}^{2}$. Then, we can express their received signals as a vector, i.e.,

(50) $$\begin{array}{cc} y^{\mathrm{LU}} & =y(\Theta_{k}^{\mathrm{LU}},\Gamma_{k}^{\mathrm{LU}})\\ \; & =H^{H}\left(\Theta^{\mathrm{LU}},\Gamma^{\mathrm{LU}}\right)x+\xi^{\mathrm{LU}}, \end{array}$$

where $\xi^{\mathrm{LU}}=[\xi_{1}^{\mathrm{LU}},\xi_{2}^{\mathrm{LU}},\cdots ,\xi_{K}^{\mathrm{LU}}{]}^{T}∼CN(0_{K\times 1},\sigma_{\xi^{\mathrm{LU}}}^{2}I_{K})$ is the AWGN vector.

Substituting Equation (20) and Equation (22) into Equation (50), the received signals can be simplified as

(51) $$\begin{array}{c} y^{\mathrm{LU}}=\sqrt{P_{t}}u+\xi^{\mathrm{LU}}, \end{array}$$

which is simply the summation of the useful information and AWGN. Then, Equation (49) can be rewritten as

(52) $$\begin{array}{c} y_{{}^{k}}^{\mathrm{LU}}=\sqrt{P_{t}}u_{k}+\xi_{k}^{\mathrm{LU}}. \end{array}$$

From Equation (52), each LU can easily recover the confidential information transmitted from the BS.

However, for the Eve with the spatial domain and the polarization domain information $\left(\Theta^{\mathrm{EVE}},\Gamma^{\mathrm{EVE}}\right)$, the received signal can be calculated by

(53) $$\begin{array}{c} y^{\mathrm{EVE}}=s^{H}\left(\Theta^{\mathrm{EVE}},\Gamma^{\mathrm{EVE}}\right)x+\xi^{\mathrm{EVE}}=\sqrt{P_{t}}s^{H}\left(\Theta^{\mathrm{EVE}},\Gamma^{\mathrm{EVE}}\right)\mathrm{Pu}+\xi^{\mathrm{EVE}}, \end{array}$$

where $\xi^{\mathrm{EVE}}=[\xi_{1}^{\mathrm{EVE}},\xi_{2}^{\mathrm{EVE}},\cdots ,\xi_{K}^{\mathrm{EVE}}{]}^{T}∼CN(0_{K\times 1},\sigma_{\xi^{\mathrm{EVE}}}^{2}I_{K})$ is the AWGN vector, $s(\Theta^{\mathrm{EVE}},\Gamma^{\mathrm{EVE}})$ is the steering vector of the Eve.

Assuming that an Eve expects to intercept the confidential information of the *k*th (k∈{1,2,⋯,K}) LU.

When the Eve is far away from the *k*th LU, we have

(54) $$\begin{array}{c} \Theta^{\mathrm{EVE}}\neq \Theta_{k}^{\mathrm{LU}},k\in \{1,2,\cdots ,K\}. \end{array}$$

Then, regardless of whether or not $\Gamma^{\mathrm{EVE}}=\Gamma_{k}^{\mathrm{LU}}$, k∈{1,2,⋯,K}, we have

(55) $$\begin{array}{c} s^{H}\left(\Theta^{\mathrm{EVE}},\Gamma^{\mathrm{EVE}}\right)P\neq e_{k}. \end{array}$$

Obviously, we can obtain that

(56) $$\begin{array}{c} y_{k}^{\mathrm{EVE}}\neq \sqrt{P_{t}}u_{k}+\xi_{k}^{\mathrm{EVE}}. \end{array}$$

Therefore, we can come to a conclusion that the Eve cannot recover the confidential messages when it is far away from the LU.

Next, we consider that an Eve is located at the same position as the *k*th LU’s as the worst situation. Here, we have

(57) $$\begin{array}{c} \Theta^{\mathrm{EVE}}=\Theta_{k}^{\mathrm{LU}},k\in \{1,2,\cdots ,K\}. \end{array}$$

In this scenario, the security can also be guaranteed for our method due to the introduction of polarization information. The Eve has no *a*-*prior* knowledge of the PS $\Gamma_{k}^{\mathrm{LU}}$ for the *k*th user, i.e., $\Gamma^{\mathrm{EVE}}\neq \Gamma_{{}^{k}}^{\mathrm{LU}}$, k∈{1,2,⋯,K}. Then, we still have

(58) $$\begin{array}{c} s^{H}\left(\Theta^{\mathrm{EVE}},\Gamma^{\mathrm{EVE}}\right)P=s^{H}\left(\Theta^{\mathrm{LU}},\Gamma^{\mathrm{EVE}}\right)P\neq e_{k}. \end{array}$$

The received signal for the Eve is given by

(59) $$\begin{array}{cc} y_{k}^{\mathrm{EVE}} & =\sqrt{P_{t}}s^{H}\left(\Theta^{\mathrm{EVE}},\Gamma^{\mathrm{EVE}}\right)\mathrm{Pu}+\xi^{\mathrm{EVE}}\\ \; & =\sqrt{P_{t}}s^{H}\left(\Theta^{\mathrm{LU}},\Gamma^{\mathrm{EVE}}\right)\mathrm{Pu}+\xi^{\mathrm{EVE}}\\ \; & \neq \sqrt{P_{t}}u_{k}+\xi_{k}^{\mathrm{EVE}}. \end{array}$$

Therefore, the Eve still cannot recover the confidential information even if it is located at the same position as the *k*th LU’s. Therefore, PLS is significantly enhanced.

### 5.4. Metrics

SER, secrecy rate, and robustness are three key measurements to evaluate the security performance of DM systems [40,41,42]. Next, the SER, secrecy rate, and robustness of the proposed DM scheme will be analyzed.

*A. SER*

For clarity, without loss of generality, assuming that all AWGNs have the same distribution with zero mean and variance $\sigma_{\xi }^{2}$ throughout the article, i.e., $\sigma_{\xi^{\mathrm{LU}}}^{2}=\sigma_{\xi^{\mathrm{EVE}}}^{2}=\sigma_{\xi }^{2}$. Meanwhile, all the transmitted baseband symbols are normalized. Therefore, using Equation (52), the signal-to-noise ratio (SNR) of the *k*th LU can be obtained by

(60) $$\begin{array}{c} r_{k}^{\mathrm{LU}}=\frac{P_{t}}{\sigma_{\xi }^{2}}. \end{array}$$

By contrast, the SNR of the conventional AN-aided DM scheme can be expressed as

(61) $$\begin{array}{c} r^{\mathrm{AN}}=\frac{\beta^{2}P_{t}}{\sigma_{\xi }^{2}}=\beta^{2}r_{k}^{\mathrm{LU}}, \end{array}$$

where β (0<β<1) is the power allocation factor for the confidential information.

Meanwhile, the received signals of the Eve are scrambled by the eavesdropping steering matrix $s(\Theta^{\mathrm{EVE}},\Gamma^{\mathrm{EVE}})$ in both the spatial and the polarized domains. By means of Equation (53), the SNR of the Eve which wants to intercept the useful information of the *k*th LU can be calculated by

(62) $$\begin{array}{c} r_{k}^{\mathrm{EVE}}=\frac{P_{t}|s_{k}^{H}\left(\Theta^{\mathrm{EVE}},\Gamma^{\mathrm{EVE}}\right)P_{k}{|}^{2}}{\sigma_{\xi }^{2}}. \end{array}$$

In this article, binary phase shift keying (BPSK) and quadrature phase shift keying (QPSK) modulation will be utilized by the proposed DM scheme. The theoretical closed-form expressions for the SER of BPSK and QPSK modulated signals over the AWGN channel can be given by

(63) $$\begin{array}{c} P_{e,BPSK}=1/2\cdot erfc\sqrt{r}, \end{array}$$

and

(64) Pe,QPSK=1−[1−11−[1−122·erfcr]2,

respectively, where *r* denotes the SNR per bit, and erfc(x)=22ππ·∫x∞e−t2dt is the complementary error function.

Therefore, using Equation (60) to Equation (64), the related SER of the proposed DM scheme can be easily calculated.

*B. Secrecy Rate*

By means of Equation (52), the signal-to-interference-plus-noise ratio (SINR) of the *k*th LU can be expressed as

(65) $$\begin{array}{c} \chi_{k}^{\mathrm{LU}}=\frac{P_{t}}{\sigma_{\xi }^{2}}=r_{k}^{\mathrm{LU}}. \end{array}$$

Since the normalized narrow-band AWGN channel is assumed, using Equation (65), the achievable secure rate of the link from the transmitter to the *k*th LU can be calculated by

(66) $$\begin{array}{c} R_{k}^{\mathrm{LU}}=\log_{2}\left(1+\chi_{k}^{\mathrm{LU}}\right). \end{array}$$

According to Equation (53), the SINR of the Eve which expects to intercept the confidential information of the *k*th LU can be given by

(67) $$\begin{array}{c} \chi_{k}^{\mathrm{EVE}}=\frac{P_{t}|s_{k}^{H}\left(\Theta^{\mathrm{EVE}},\Gamma^{\mathrm{EVE}}\right)P_{k}{|}^{2}}{P_{t}\sum_{l=1,l\neq k}^{K}|s_{l}^{H}\left(\Theta^{\mathrm{EVE}},\Gamma^{\mathrm{EVE}}\right)P_{l}{|}^{2}+\sigma_{\xi }^{2}}. \end{array}$$

In the light of (67), the achievable rate of the link between the base station and the Eve can be obtained as

(68) $$\begin{array}{c} R_{k}^{\mathrm{EVE}}=\log_{2}\left(1+\chi_{k}^{\mathrm{EVE}}\right). \end{array}$$

Therefore, the secrecy rate of the proposed DM scheme can be defined as [3,40]

(69) $$\begin{array}{c} R=\sum_{k=1}^{K}{\left[R_{k}^{\mathrm{LU}}-R_{k}^{\mathrm{EVE}}\right]}^{†}, \end{array}$$

where ${\left[\cdot \right]}^{†}$ refers to max(0,·).

*C. Robustness*

In this part, the robustness of the proposed DM scheme including the impacts of imperfect estimation of the LUs’ directions and the depolarization effect of the received signals will be investigated.

■ Imperfect Estimation of the LUs’ Directions 

In the proposed DM scheme, the directions of *K* LUs are assumed to be a-priori known for the BS. However, in practical applications, the directions of the LUs are usually obtained by utilizing high-resolution direction of arrival (DOA) estimation algorithms such as [43,44,45]. Even though, there still exits an estimated error in the directional information inevitably.

The estimated directional angle of the *k*th LU can be assumed as

(70) $$\begin{array}{c} {\hat{\Theta }}_{k}^{\mathrm{LU}}=\Theta_{k}^{\mathrm{LU}}+\Delta \Theta_{k}^{\mathrm{LU}}, \end{array}$$

where $\Delta \Theta_{k}^{\mathrm{LU}}$ is the estimated angle error.

The estimated angle error will impose adverse impact on the precoding matrix P, which will severely degrade the security performance of the proposed DM scheme. In this case, the precoding matrix with estimation errors should be replaced by

(71) $$\begin{array}{c} \hat{P}={\left[H\left({\hat{\Theta }}^{\mathrm{LU}},\Gamma^{\mathrm{LU}}\right)\right]}^{+}, \end{array}$$

where $H({\hat{\Theta }}^{\mathrm{LU}},\Gamma^{\mathrm{LU}})$ is the estimated steering matrix composed by the *K* estimated steering vectors, which is given by

(72) $$\begin{array}{c} H\left({\hat{\Theta }}^{\mathrm{LU}},\Gamma^{\mathrm{LU}}\right)=\left[s\left({\hat{\Theta }}_{1}^{\mathrm{LU}},\Gamma_{1}^{\mathrm{LU}}\right),\cdots ,s\left({\hat{\Theta }}_{k}^{\mathrm{LU}},\Gamma_{k}^{\mathrm{LU}}\right),\cdots ,s\left({\hat{\Theta }}_{K}^{\mathrm{LU}},\Gamma_{K}^{\mathrm{LU}}\right)\right]. \end{array}$$

According to Equation (72), it is not hard to see that the normalization characteristic in Equation (21) will be affected by the estimation errors. Simulations will be provided in next Section to demonstrate that the security of the proposed DM scheme can also be guaranteed as long as the estimation errors of the LUs’ direction are in an acceptable range. In practical applications, some robust DM synthesis methods can be used to fight against the imperfect estimations of the LUs’ directions [25,46].

■ Depolarization Effect of the Received Signals

In time-varying multi-path channels, the depolarization effect of the wireless channel mainly includes polarization mode dispersion (PMD) and polarization-dependent loss (PDL). While the PMD effect will cause different damage to the PDL effect suffered by the DM scheme on different carriers. For the proposed single-carrier wireless communication in this article, the carrier frequency is much larger than the bandwidth, so the frequency difference within the bandwidth range is small. Therefore, the PMD effect is not obvious. The PDL effect is the main factor that impairs the performance of the DM scheme.

The degree of depolarization effect of wireless channels is determined by the difference in the eigenvalues of the channel matrix. In order to reduce or eliminate the influence of the wireless channel depolarization effect on a DM system, the degree of difference in the eigenvalues of the channel matrix should be reduced as much as possible. To this end, when the scatterers between transmitter and receiver depolarize the signals, this issue can be effectively solved by pre-compensating the polarization distortion of the transmitted signals at the transmitting side. It is assumed that the transmitter can obtain perfect channel state information (CSI), and the CSI remains unchanged during the channel coherence time. Based on the CSI obtained, the transmitting polarization state can be pre-compensated, i.e., multiplying each polarization state by a pre-compensation matrix. Some other depolarization suppression methods can be used to fight against the depolarization effect of the received signals [47,48].

## 6. Simulations and Discussions

In this section, we will provide several design examples based on a 10λ uniform linear PSA with a half-wavelength inter-element spacing to verify the SER performance of the proposed DM scheme. Then, the secrecy rate, and robustness of the proposed DM scheme are simulated.

Without loss of generality, we assume that the azimuth angles are fixed $\phi =90^{\circ }$ for all design examples. $10^{7}$ random symbols are used in the SER simulations, the AWGN power is the same for all directions, and signal-to-noise-ratio (SNR) = 12 dB.

### 6.1. SER

*A. Single beam with fixed polarization information*

First, we will consider broadside and off-broadside design examples for single beam to transmit two independent data streams with different PSs to verify that the introduction of PS can increase the spectral efficiency. That can also be viewed as the polarization multiplexing. The capacity performance of our method is equal to the polarization multiplexing that two separate signals are transmitted by a PSA. We assume that the signals are Gray-coded QPSK modulated. The PSs are defined by $\Theta_{1}=\left(45^{\circ },90^{\circ }\right)$ for data stream 1 for a left-hand circular polarization, and $\Theta_{2}=\left(45^{\circ },-90^{\circ }\right)$ for data stream 2 for a right-hand circular polarization at arbitrary directions. For each data stream, the desired beam pattern is a value of one with 90${}^{\circ }$ phase shift at the desired mainlobe, i.e., symbols “00”, “01”, “11”, “10” correspond to 45${}^{\circ }$, 135${}^{\circ }$, −135${}^{\circ }$, −45${}^{\circ }$. Meanwhile, the desired beam patterns over the sidelobe region are random complex numbers with the amplitudes being approximate zero.

For broadside design example, the desired direction is set $\theta_{ML}=0^{\circ }$; for off-broadside design example, the desired direction is set $\theta_{ML}=30^{\circ }$. The sidelobe regions are sampled every 1${}^{\circ }$ except the mainlobe direction.

**Figure 6:**
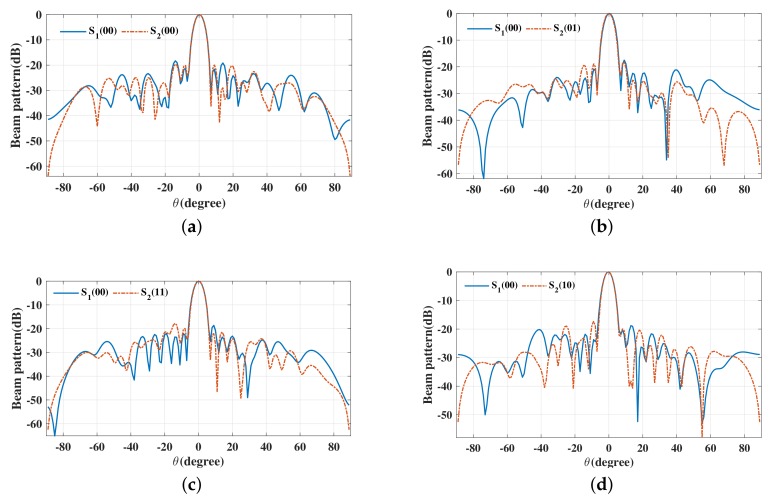
Beam patterns for broadside $\theta_{ML}=0^{\circ }$ for single beam for symbol pairs (**a**) “00,00”, (**b**) “00,01”, (**c**) “00,11”, (**d**) “00,10”.

**Figure 7:**
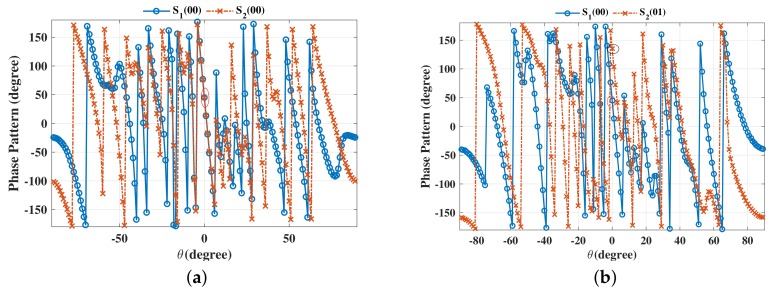

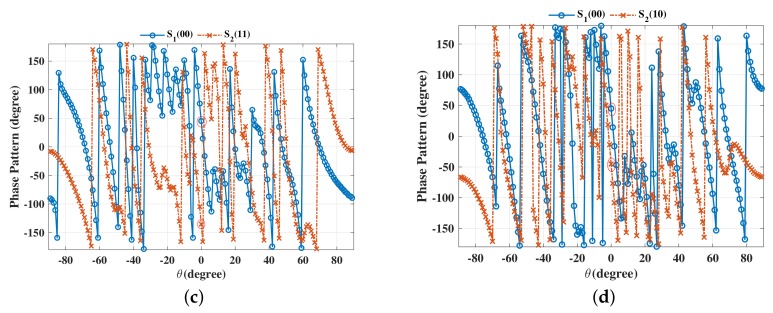
Phase patterns for broadside $\theta_{ML}=0^{\circ }$ for single beam for symbol pairs (**a**) “00,00”, (**b**) “00,01”, (**c**) “00,11”, (**d**) “00,10”.

**Figure 8:**
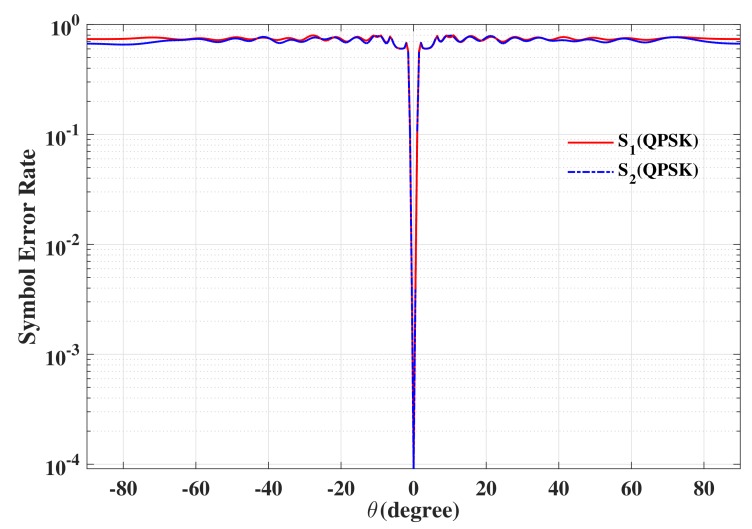
The resulting symbol error rate (SER) curve for broadside $\theta_{ML}=0^{\circ }$ for two data streams.

The beam patterns for broadside for symbol pairs “00,00”, “00,01”, “00,11” and “00,10” are depicted in Figure 6, where all main beams are exactly pointed to 0${}^{\circ }$ with normalized magnitude 0 dB level, denoting that the amplitude of the desired data streams as expected. The phase patterns for broadside for those symbol pairs are shown in Figure 7. It is not hard to see that the phases of the two data streams in the desired direction are in line with the standard QPSK constellation, while in the sidelobe regions, phases are random enough. The beam and phase patterns for other twelve symbol pairs are not displayed here on account of the similar features as the four symbol pairs mentioned above. The resulting SER curves are demonstrated in Figure 8. It is indicated that the low SER is achieved in the desired direction, while in other undesired directions, the SER approximates the upper bound of a QPSK transmission system (0.75), representing that the directional modulation has been realized effectively.

**Figure 9:**
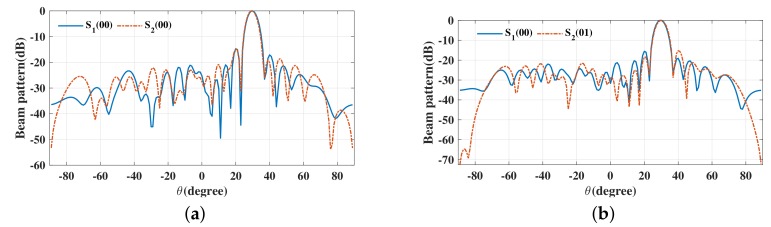

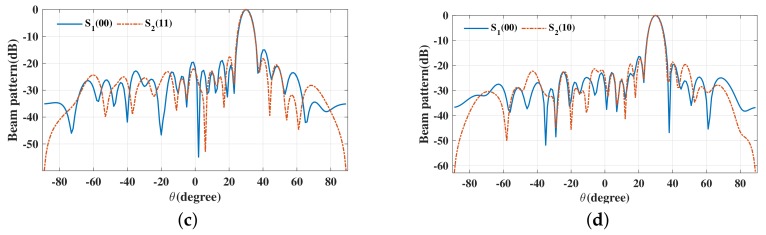
Beam patterns for broadside $\theta_{ML}=30^{\circ }$ for single beam for symbol pairs (**a**) “00,00”, (**b**) “00,01”, (**c**) “00,11”, (**d**) “00,10”.

**Figure 10:**
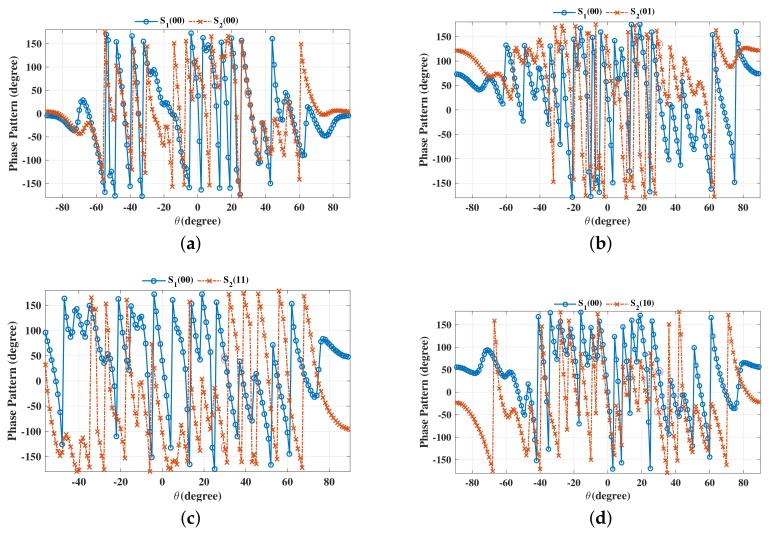
Phase patterns for broadside $\theta_{ML}=30^{\circ }$ for single beam for symbol pairs (**a**) “00,00”, (**b**) “00,01”, (**c**) “00,11”, (**d**) “00,10”.

**Figure 11:**
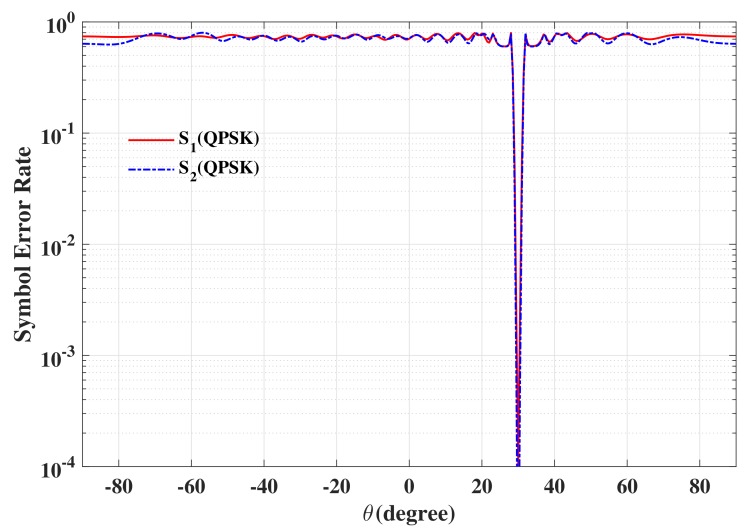
The resulting SER curve for off-broadside $\theta_{ML}=30^{\circ }$ for two data streams.

The beam and phase patterns for off-broadside $\theta_{ML}=30^{\circ }$ for symbol pairs “00,00”, “00,01”, “00,11” and “00,10” are depicted in Figure 9 and Figure 10, respectively. The resulting SER curves are displayed in Figure 11. It is easy to see that the designed responses are slightly less desirable because the wider mainlobe. Even so, the PLS can still be enhanced.

*B. Multiple beams with fixed polarization information*

**Figure 12:**
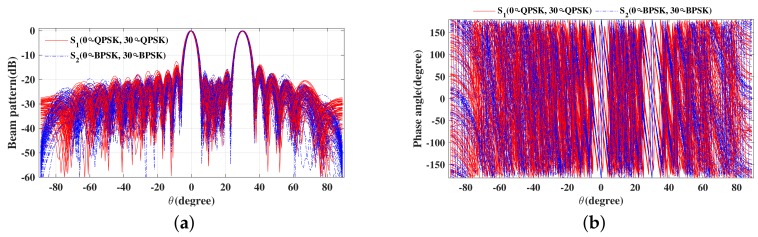
The simulated far-field (**a**) magnitude patterns and (**b**) phase patterns for 50 symbols.

Next, we will consider design examples for multiple beams to transmit different or the same modulation information. Take two beams for example, one data stream transmits the QPSK modulation symbols in two desired directions with a horizontal polarization $\Theta_{1}=\left(0^{\circ },0^{\circ }\right)$, and another data stream transmits the BPSK modulation symbols in the same two directions with a vertical polarization $\Theta_{1}=\left(90^{\circ },0^{\circ }\right)$. The simulated far-field (a) magnitude patterns and (b) phase patterns for 100 random symbols are shown in Figure 12.

**Figure 13:**
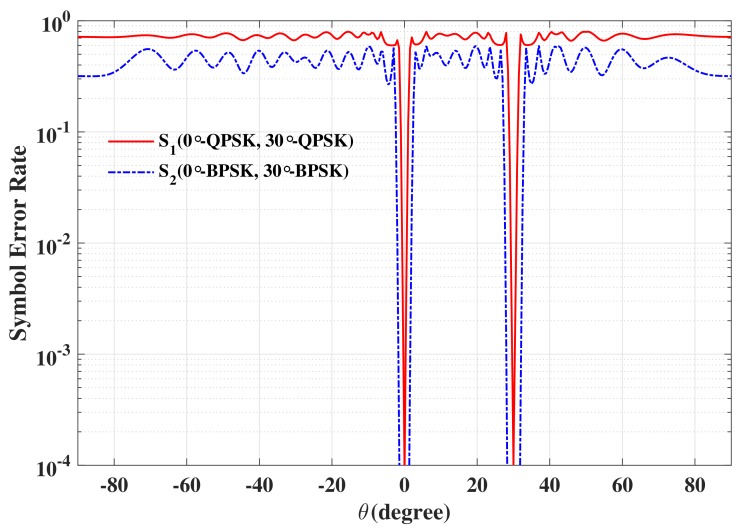
The resulting SER curve versus elevation angle for two data streams in two desired directions.

Thus, from Figure 12, it can be observed that standard QPSK and BPSK constellation patterns are only along the prescribed directions, $0^{\circ }$ and $30^{\circ }$ as expected, with the signal IQ formats along all other directions being distorted, in such a manner to lower the possibility of interception by eavesdroppers located in these regions. Figure 13 shows the SER performance versus elevation angle for the two data streams transmitted when SNR equals 12 dB. It is obvious to find that the SER performance of the two data streams is the same as the traditional QPSK or BPSK signal at desired directions ($0^{\circ }$ and $30^{\circ }$), while the SER performance is deteriorated seriously when the elevation angle is off the desired directions. Therefore, the channel capacity is double increased, and this characteristic of the designed signals is also beneficial for PLS enhancement.

*C. Multiple beams with variable polarization information*

**Figure 14:**
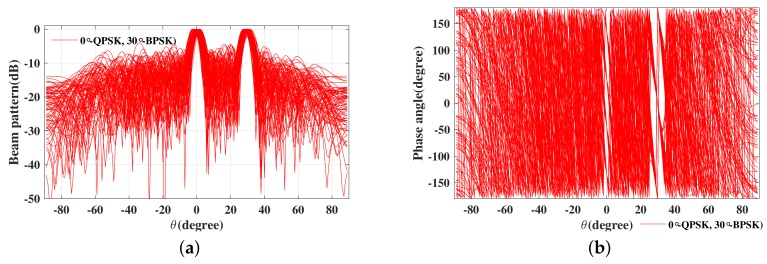
The simulated far-field (**a**) magnitude patterns and (**b**) phase patterns for 50 symbols with variable polarization information.

In the following design example, it is assumed that a signal stream modulated with QPSK are projected along broadside $0^{\circ }$, while another independent data stream modulated with BPSK, is transmitted along off-broadside $30^{\circ }$ by a 21-element uniform linear PSA. Meanwhile, we designate the polarization $\Theta_{1}=\left(45^{\circ },45^{\circ }\right)$ at the direction $0^{\circ }$, the polarization $\Theta_{2}=\left(45^{\circ },-45^{\circ }\right)$ at the direction 30${}^{\circ }$, and the PSs are generated randomly at other undesired directions by the polarization control unit in Figure 3. The simulated far-field (a) magnitude patterns and (b) phase patterns for 100 random symbols are shown in Figure 14.

**Figure 15:**
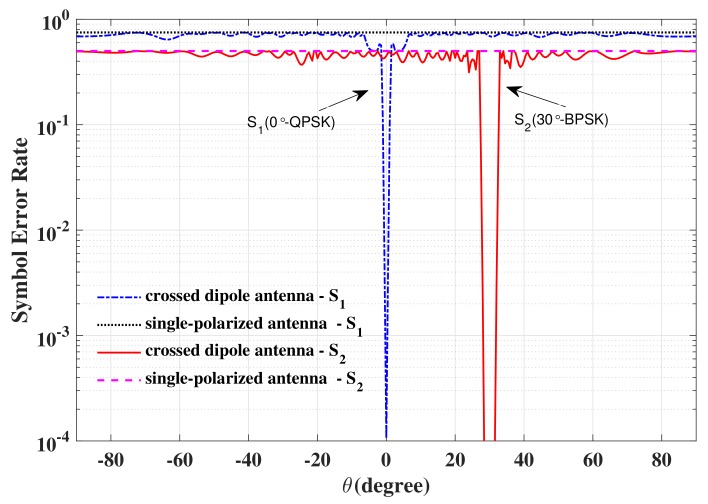
The SER simulation results versus elevation angle obtained for receivers using a polarization sensitive antenna or a single-polarized antenna.

**Figure 16:**
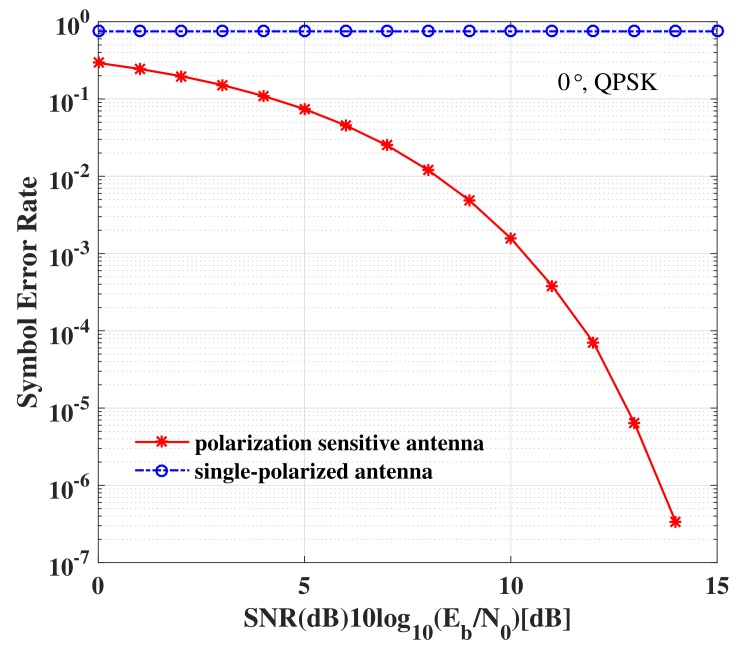
The SER performance versus SNR for receivers at the desired direction $0^{\circ }$ utilizing a polarization sensitive antenna or a single-polarized antenna.

In Figure 15, the SER simulation results versus elevation angle obtained for receivers using a polarization sensitive antenna and a single-polarized antenna are depicted. Two observations can be generalized from Figure 15: (1) the SERs of the LUs using a polarized sensitive antenna at the desired directions ($0^{\circ }$ and $30^{\circ }$) are as good as the ideal case; (2) No matter where the Eve is located, it cannot intercept the exact confidential information using a single-polarized antenna due to the high SER. Further, the SER performance versus SNR for receivers at the desired direction $0^{\circ }$ utilizing a polarization sensitive antenna and a single-polarized antenna is displayed in Figure 16. From Figure 16, we can see that even if the eavesdroppers locate in the desired directions with high SNR enough, the confidential information still cannot be demodulated exactly, when the eavesdroppers use the antennas different from the LUs, i.e., $\Gamma^{\mathrm{EVE}}\neq \Gamma_{{}^{k}}^{\mathrm{LU}}$, k∈{1,2,⋯,K}. Therefore, the DM technique based on a PSA is an effective approach to enhance PLS.

### 6.2. Secrecy Rate

**Figure 17:**
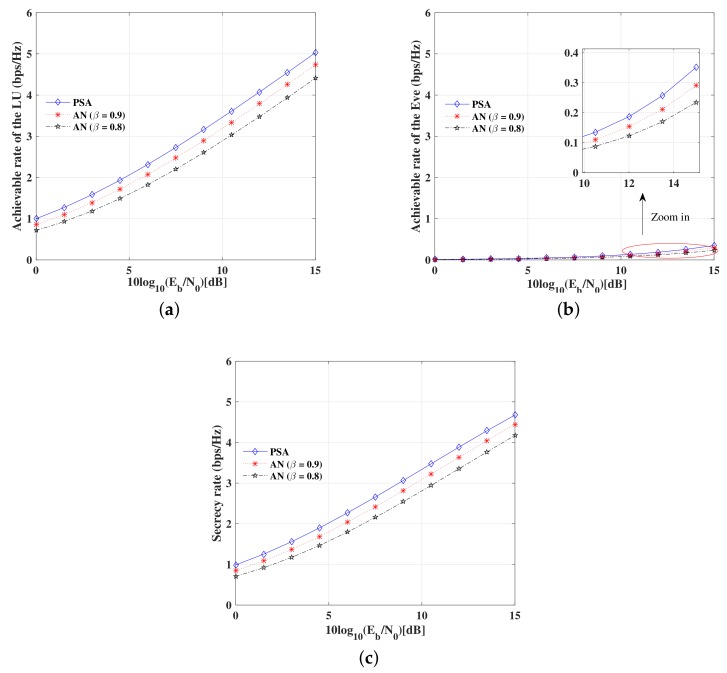
Secrecy rate performance of the proposed DM scheme and the conventional AD-aided DM schemes. (**a**) Achievable rate of the LU versus SNR; (**b**) Achievable rate of the Eve versus SNR; (**c**) Secrecy rate of the system.

Figure 17a depicts the achievable rate versus SNR per bit of the LU for the proposed DM scheme and the conventional AN-aided DM schemes. As was expected, the achievable rates of the LU for the proposed DM scheme are much higher than that of the conventional AN-aided DM schemes, especially when the power allocation factor β of the AN-aided DM schemes is smaller.

Figure 17b illustrates the achievable rate versus SNR per bit of the Eve for the proposed DM scheme and the conventional AN-aided DM schemes. As might have been expected, the achievable rates are approximate to 0 bps/Hz when the Eve is not in the desired directions. It is observed that the achievable rates of the Eve for the proposed DM scheme is a little higher than that of the conventional AN-aided DM schemes.

It is demonstrated by Figure 17a,b that, due to without AN, the proposed DM scheme is more power-efficient than the traditional AN-aided DM schemes.

Furthermore, Figure 17c shows the secrecy rate versus SNR per bit for the proposed DM scheme and the conventional AN-aided DM schemes. Whatever the power allocation factor β takes, the secrecy rates of the LU for the proposed DM scheme are much higher than that of the conventional AN-aided DM schemes with a fixed normalized total transmitting power. Meanwhile, the secrecy rates of the proposed DM scheme are always positive wherever the Eve is located. Therefore, the proposed DM scheme is very secure and reliable.

### 6.3. Robustness

**Figure 18:**
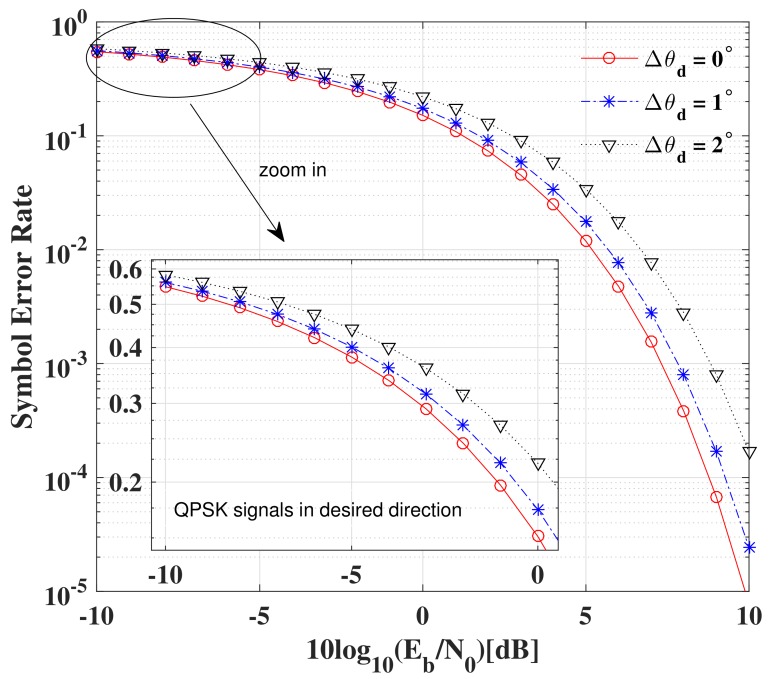
System model.

The SER performance versus SNR per bit with different imperfect estimation errors of the LU’s directions is shown in Figure 18. For clarity, only the SER curves for one LU with QPSK modulation are presented, from which the same conclusions can also hold for other LUs.

It is not difficult to observed from Figure 18 that there is only about 0.5 dB loss of SNR when the estimated direction angle error is $\Delta \theta_{d}=1^{\circ }$ for a given SER performance (e.g., $SER=10^{-4}$) compared with the ideal case with no errors. Obviously, as long as the estimated angle errors are less than $\Delta \theta_{d}=1^{\circ }$, at most 0.5 dB additional SNR is required to achieve the same SER as the ideal case. Therefore, in order to achieve secure and reliable DM transmission for all LUs, the estimated direction errors for all LUs should be less than $\Delta \theta_{d}=1^{\circ }$.

## 7. Conclusions

DM technique based on a PSA has been proposed and further studied for PLS enhancement from the sampling perspective in spatial and polarization domain and from the signal processing perspective, respectively. We have formulated a design example to send two different independent data streams simultaneously at the same desired directions, same frequency, but with different PSs. It indicates that the channel capacity can be easily increased by the introduction of polarization information. Meanwhile, we have formulated another design example to send two different independent data streams simultaneously at two desired directions, with two fixed PSs, respectively, and off the desired directions, the PSs are distorted randomly. Simulation results verify that the security performance is significantly enhanced.

## Figures and Tables

**Table 1:** Notations used throughout the article.

Symbols	Usage	Symbols	Usage
${\left(\cdot \right)}^{T}$	Transpose operator	${\left(\cdot \right)}^{H}$	Complex conjugate transpose operator
${\left(\cdot \right)}^{-1}$	Inverse operator	${\left(\cdot \right)}^{+}$	Moore-Penrose pseudo inverse operator
$\left|\cdot \right|$	Modulus operator	${∥\cdot ∥}_{2}$	$l_{2}$-norm operator
⊗	Kronecker product operator	Π	Quadrature operator
Σ	Sum operator	erfc(·)	Complementary error function
Ξ(·)	Phase acquisition function	CN(·,·)	Standard normal distribution
max(·,·)	Returns the largest element	${\left[\cdot \right]}^{†}$	max(·,0)
R,C	Real number, complex number	$I_{K}$	Identity matrix with size K×K

## Appendix A

In this appendix, the derivation of the optimal solution for the optimization problem Equation (16) will be given. For simplicity, we omit the subscript *i*. Meanwhile, the optimization problem can be equivalently reformulated as

(A1) $$\begin{array}{c} \min\;\;\;{∥p_{SL}-w^{H}S_{SL}∥}_{{}_{2}}^{2}\\ s.t.p_{ML}=w^{H}S_{ML}. \end{array}$$

According to the method of Lagrange multipliers, first, we let

(A2) $$L\left(w,\mu \right)={∥p_{SL}-w^{H}S_{SL}∥}_{{}_{2}}^{2}+\mu \left(w^{H}S_{ML}-p_{ML}\right).$$

Then, to find the partial derivatives of function L(w,μ) with respect to w and μ, respectively.

(A3) $$\frac{\partial L(w,\mu )}{\partial w}=-2\left(p_{SL}-w^{H}S_{SL}\right)S_{{}_{SL}}^{H}+\mu S_{{}_{ML}}^{H},$$

(A4) $$\frac{\partial L(w,\mu )}{\partial \mu }=w^{H}S_{ML}-p_{ML}.$$

Next, let $\frac{\partial L(w,\mu )}{\partial w}=0$, we obtain

(A5) $$w^{H}=\frac{1}{2}\left(2p_{SL}S_{{}_{SL}}^{H}-\mu S_{{}_{ML}}^{H}\right){\left(S_{SL}S_{{}_{SL}}^{H}\right)}^{-1}.$$

Let $\frac{\partial L(w,\mu )}{\partial \mu }=0$, and inserting Equation (A5) into Equation (A4), we obtain

(A6) $$\mu =2(p_{SL}S_{{}_{SL}}^{H}{\left(S_{{}_{ML}}^{H}\right)}^{-1}-p_{ML}S_{{}_{ML}}^{-1}S_{SL}S_{{}_{SL}}^{H}{\left(S_{{}_{ML}}^{H}\right)}^{-1}).$$

Finally, inserting Equation (A6) into Equation (A3), we obtain the optimal solution w for the optimization problem Equation (A1), also for the optimization problem Equation (16), which is given by

(A7) $$\begin{array}{c} w={\left(S_{SL}S_{{}_{SL}}^{H}\right)}^{-1}\left(S_{SL}p_{{}_{SL}}^{H}-S_{ML}{\left(S_{{}_{ML}}^{H}{\left(S_{SL}S_{{}_{SL}}^{H}\right)}^{-1}S_{ML}\right)}^{-1}\left(S_{{}_{ML}}^{H}{\left(S_{SL}S_{{}_{SL}}^{H}\right)}^{-1}S_{SL}p_{{}_{SL}}^{H}-p_{{}_{ML}}^{H}\right)\right). \end{array}$$

Let $R=S_{SL}S_{{}_{SL}}^{H}$, then, Equation (A7) can be simplified as

(A8) $$\begin{array}{c} w=R^{-1}\left(S_{SL}p_{{}_{SL}}^{H}-S_{ML}{\left(S_{{}_{ML}}^{H}R^{-1}S_{ML}\right)}^{-1}\left(S_{{}_{ML}}^{H}R^{-1}S_{SL}p_{{}_{SL}}^{H}-p_{{}_{ML}}^{H}\right)\right). \end{array}$$

The proof is completed.
